# Decision-making in information seeking on texts: an eye-fixation-related potentials investigation

**DOI:** 10.3389/fnsys.2013.00039

**Published:** 2013-08-14

**Authors:** Aline Frey, Gelu Ionescu, Benoit Lemaire, Francisco López-Orozco, Thierry Baccino, Anne Guérin-Dugué

**Affiliations:** ^1^Chart-Lutin, Université Paris 8Saint-Denis, France; ^2^GIPSA-lab, University of Grenoble AlpesGrenoble, France; ^3^Laboratoire de Psychologie et NeuroCognition, University of Grenoble AlpesGrenoble, France

**Keywords:** information seeking, eye-fixation-related potentials, semantic processing, decision-making, EEG, eye movements

## Abstract

Reading on a web page is known to be not linear and people need to make fast decisions about whether they have to stop or not reading. In such context, reading, and decision-making processes are intertwined and this experiment attempts to separate them through electrophysiological patterns provided by the Eye-Fixation-Related Potentials technique (EFRPs). We conducted an experiment in which EFRPs were recorded while participants read blocks of text that were semantically highly related, moderately related, and unrelated to a given goal. Participants had to decide as fast as possible whether the text was related or not to the semantic goal given at a prior stage. Decision making (stopping information search) may occur when the paragraph is highly related to the goal (positive decision) or when it is unrelated to the goal (negative decision). EFRPs were analyzed on and around typical eye fixations: either on words belonging to the goal (target), subjected to a high rate of positive decisions, or on low frequency unrelated words (incongruent), subjected to a high rate of negative decisions. In both cases, we found EFRPs specific patterns (amplitude peaking between 51 to 120 ms after fixation onset) spreading out on the next words following the goal word and the second fixation after an incongruent word, in parietal and occipital areas. We interpreted these results as delayed late components (P3b and N400), reflecting the decision to stop information searching. Indeed, we show a clear spill-over effect showing that the effect on word N spread out on word N + 1 and N + 2.

## Introduction

Seeking information in a newspaper or on a web page, both composed of multiple blocks of text demands that rapid decisions be made about whether to stop the reading of the current block and switch to another one. Quite often, the time constraint is strong and texts are not entirely processed. People are able to judge from a couple of words whether they found a text relevant or not. This involves two concurrent cognitive processes, (1) the word-to-word collection of the necessary and relevant information and, (2) the decision to leave the current block once provided with enough information. *Reading and decision-making* are therefore processes which intertwine during the search for information. For instance, it has been demonstrated that in situations where they need to solve problems, people decide to stop seeking information by estimating the cost of information with regards to the environment in which the task is performed. The general behavior is found to be sensitive to even the smallest changes in information-seeking costs. However, in the case of reading, costs are more difficult to determine since the goal is ill-defined and mostly based on semantic processing. To develop a cognitive model adapted to the search for information, it is necessary to feed it with human variables sensitive to *semantic processing* and *continuously available* throughout the progression on the page.

On the other hand, the search for information on textual web pages constantly requires that the reader switches between different *strategies* (reading, searching, stopping rereading) alternating from deep reading to word searching. Carver (1990) identified five reading strategies based on the reader's goal: memorizing, learning, rauding, skimming, and scanning. He assumed that these strategies might be clustered by the reading rates (in words per min). Hence, the reading strategy (called *rauding*) is achieved on an average 300 Wpm while scanning is performed at 600 Wpm and used when readers are looking for a particular word. Our task is situated in between these strategies and corresponds to what Carver called *skimming* (450 Wpm). Recent simulations using scanpaths as human metrics have been developed for an automatic identification of some of these strategies and they show the moment by moment orientation of attention but once again no completely reliable information on semantic processing was provided with this metric.

Consequently, the main issue of this paper is to distinguish between semantic and decision-making processes in information-seeking tasks, through the joint analysis of eye-tracking and EEG data (the so-called Eye-Fixation-Related Potentials—EFRPs) (Hutzler et al., 2007; Baccino, 2011; Dimigen et al., 2011; Kliegl et al., 2012). We used this technique because not every metric (eye movements and EEG) is able to unveil on its own what is really happening during the search for information. Eye-tracking data provides highly valuable information on the sequence of words that have been fixated by the reader before he/she decides to stop reading. Fixation durations are also available, but they are a weak indicator of what happens during the reading process, because several factors influence the fixation duration (for example, word frequency or word predictability) even if no decision-making is involved. In addition, there is no one-to-one temporal mapping between a fixation on a word and the cognitive processes associated with that word: word processing may continue after the reader's have left the word. For instance, this well-known *spill-over effect* is demonstrated by the fact that a low-frequency word results in an extra processing time, not only for that word but also for the next one (Rayner and Duffy, 1986). Knowing when a word has been fixated is therefore insufficient to know exactly when this word is processed (Rayner, 1978). Finally, most often the decision to stop searching does not occur within the last milliseconds before the participant leaves the current text, for some time elapses between these two events. People need extra time before they move their eyes away from the current text. Therefore, decision-making is not necessarily associated with the last fixation.

Similarly, EEG data do not provide enough information on their own either. The reason is threefold: (1) it is impossible to know exactly which words have been fixated during a real reading task, (2) consequently, in EEG experiments on reading, words have to be presented one at a time onto the screen at a speed of about one word per second, (3) the EEG signal is “contaminated” by saccades.

However, the EEG technique has allowed pointing out stereotyped electrophysiological responses to specific cognitive events. In particular, it has been shown that an element that is unexpected in this context elicits a larger negative waveform distributed over the centro-parietal areas and occurring about 400 ms after the stimulus onset. This so-called N400 component was first identified by Kutas and Hillyard (1980) and is usually associated with tasks of visual and auditory comprehension of sentences, in which the amplitude of the N400 is correlated with the degree of incongruence of the sentence and final word (Key et al., 2005). More specifically, the incongruous words elicited a larger amplitude of the N400 response than the congruous ones (e.g., *The man liked his coffee with dog* elicits a larger N400 amplitude than *The man liked his coffee with sugar*). The N400 amplitude also inversely correlates with cloze-probability levels, defined as, for an item, the percentage of people that will continue a sentence fragment with that particular item (Gonzalez-Marquez et al., 2007). Therefore, it was proposed that the N400 reflects processes of semantic priming or activation (Kutas and Hillyard, 1984)—i.e., the easy integration of a word into a context, or the extent to which the context pre-activates the word - to reflect the system expectation for either a content word or an index of processing difficulty. Later, meaningful stimuli other than words, such as faces (Barrett and Rugg, 1989), pictorial stimuli (Barrett and Rugg, 1990; Praterelli, 1994), objects (Ganis and Kutas, 2003), or music (Koelsch et al., 2004) were found to elicit N400-like potentials. In brief, N400 seems to reflect the degree of contextual facilitation and semantic context integration (DeLong et al., 2011). Lastly, some recent consideration has been given to the possibility that the N400 potential indexes activation processes, but also inhibition processes (see Debruille, 2007 for a review).

Another component that has been extensively studied is the P300 component. P300 is a positive component that develops over parietal areas when a subject detects an infrequent stimulus, expected yet unforeseeable, through a series of stimuli (Sutton et al., 1965). The experimental protocol that revealed this component, i.e., the “oddball paradigm,” consists in successively presenting two types of stimuli that differ in one of their physical parameters (e.g., for auditory stimuli, two sounds with different pitches) with different probabilities of occurrence: one is frequent (e.g., 80% of trials), the other is rare (e.g., 20% of trials). Note that it is not the parameters of the physical stimulation that determine the appearance of P300, but their status, that is to say their probability of occurrence. In this respect, a larger P300 is elicited by the events representing the low-probability category (Donchin, 1981). To elicit P300, the participant must be actively involved in the task, either by counting or by pressing button in response to the rare stimuli. The amplitude of P300 therefore served as our covert measure of attention that arises independently of behavioral responding (Gray et al., 2004). The amplitude of P300 is proportional to the amount of attentional resources engaged in processing a given stimulus (Johnson, 1988) and is not influenced by factors relating to the selection or execution of a response (Crites et al., 1995). In addition to low probability, stimulus properties that heighten the amplitude of P300 are relevant to the subject's task (Squires et al., 1977) and qualitative deviance (Nasman and Rosenfeld, 1990). In brief, the amplitude of P300 is affected by attention, stimulus probability, stimulus relevance, and the amount of available processing resources (Key et al., 2005). The latency of P300 is assumed to reflect the duration of the stimulus evaluation. The functional interpretation of P300 consists of memory updating, active stimulus discrimination, and categorization, as well as response preparation (Donchin and Coles, 1988).

P300 has been decomposed into two components, P3a and P3b. P3a, with a shorter latency (Knight, 1996) and a more frontal distribution, occurs even when the participant passively receives stimuli and is not required to actively respond to the targets (Timsit-Berthier and Gerono, 1998). P3a may be interpreted as an attentional shift in response to an unexpected disruption in the environment (Yamaguchi and Knight, 1991), the physiological correlate of a reaction of orientation toward novelty, the reflection of involuntary attention. Unlike P3a, the unpredictability of a stimulus is insufficient to demonstrate P3b, it is therefore necessary that the participant pays attention and responds to stimulation. According to Hansen and Hillyard (1983), P3b is associated with the final decision made on the status of the stimulus with regards to the required task. In general, P3b was associated with discrimination, categorization, selection, matching processes, and decision-making (see Picton, 1992; Hruby and Marsalek, 2003 for review). P3b does not seem to directly reflect processes relating to stimulus memorization but rather the process of evaluating the stimulus inducing decision-making.

EEG and eye-tracking data are therefore complementary in the study of the reading activity.

The aim of our experiment is to investigate the EFRPs during the search for information in a text. Participants had to make binary decisions about the semantic relatedness of a text to a goal. People would decide to stop reading for two different reasons. Firstly, they would realize that the current text was related to the goal. They would not need to read further after they found what they were looking for. We call it a *positive decision*. Secondly, they would find that the text had nothing to do with the goal. We call it a *negative decision*. In both cases, people would stop reading. To simplify the EFRPs analyses, we have defined two kinds of words that are likely to trigger these decisions. We assume that positive decisions should be triggered by *target words* (i.e, words whose verbal form belongs to the search goal). For instance, if the goal is “presidential campaign,” the word “president” may trigger a positive decision. In the same way, our hypothesis is that negative decisions result from the presence of so-called *incongruent words* (i.e, words that are specific to a domain other than that of the goal). For instance, “basketball” is a word that is specific to a particular domain, which has nothing to do with “presidential campaign.” Therefore, this incongruent word is a good “candidate” for the occurrence of a negative decision.

## Methods

### Participants

This experiment lasted about 1 h and 40 minutes and involved a total of 21 participants, all French native speakers. For technical reasons, four participants could not be registered or were excluded from the final data analysis. The seventeen remaining participants (7 women and 10 men, 16 right-handed and 1 left-handed, aged 19–43 years, mean age 27 years, *SD* = 8 years) had normal or corrected-to-normal visual acuity, and had no known neurological disorder. The purpose of the study remained unknown to them. They all gave written consent and were paid 20€ for their participation in the experiment. The whole experiment was reviewed and approved by the ethics committee of the CHU (“*Centre Hospitalier Universitaire*”) of Grenoble (RCB: *n*° 2011-A00845-36).

### Textual material

First, a set of thirty goals (topic) was created. Each goal is expressed as a nominal phrase in French such as “observation des planètes” (planet observation), “réhabilitation des logements” (housing renovation), “associations humanitaires” (humanitarian associations), etc. For each goal, six texts were created in French, two of which were highly related to the goal (HR), two moderately related (MR), and two unrelated (UR). For each goal, participants had to read texts that were either highly related (2), or moderately related (2), or unrelated (2) to a given goal. Finally they had to read 180 texts: (2HR + 2MR + 2UR) × 30 goals.

This task requires a binary decision for each text. We have considered, but indeed not a posteriori verified, that if we presented only two kinds of texts (HR, and UR), the task would be too easy and the participants could answer very rapidly after very few words, and even without a linear reading. Our goal was not to design a reflex task. We wanted to design a task where ideally reading and decision-making were intertwined and this intertwining depended on both the structure of the text and the participant. Therefore, our assumption was that, without MR texts, reading, and decision-making would not be intertwined.

**Figure 1:**
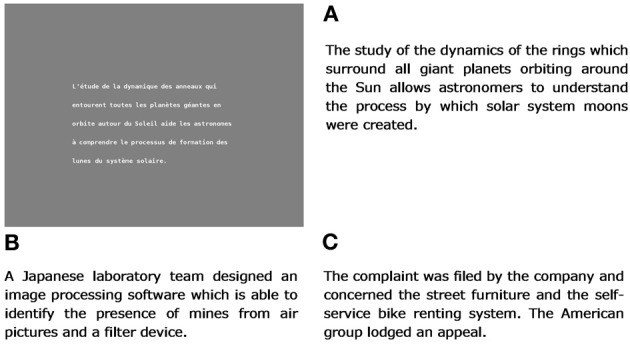
**English translation of three texts for the goal “observation des planètes” (planet observation); (A) a highly related text- the visual presentation of the French text is shown on the left-, (B) a moderately related text, and (C) an unrelated text**.

In order to control the semantic relatedness of the texts to the goals, a method called Latent Semantic Analysis (Landauer et al., 2007) was used, which consists in computing semantic similarities between texts. LSA was trained on a 24 million word French corpus composed of all the articles published in the newspaper “Le Monde” in 1999. A 300 dimension space was generated based on the corpus, by means of a singular value decomposition of the word × text occurrence matrix [see Martin and Berry (2007) for more details]. Each word of the corpus being represented by a 300 dimension vector, new texts can also be represented by a vector through the simple sum of their word vectors. A cosine function was used to compute the similarity between vectors. The higher the cosine value, the more similar the two texts are. For highly related texts, cosine with the goal was above 0.40, for moderately related texts, cosine was between 0.15 and 0.30, and for unrelated texts, cosine was below 0.10. Figure 1 shows the English translation of three examples of texts (HR, MR, and UR) for the goal “observation des planets,” as well as an example of how they were visually presented to the participants. From the point of view of analyses, the whole material was organized into three sets of 30 × 2 = 60 texts each, respectively for all the highly related, moderately related and unrelated texts.

The texts were written in “DejaVu” font. The letters were black on a medium gray background. All the texts were composed of an average 5.18 sentences (*SD* = 0.7) and 30.1 words (*SD* = 2.9). Each word was composed of an average 5.34 characters (*SD* = 3.24). The average number of lines was 5.18 (*SD* = 0.68). In average, the text was displayed with 40.1 (*SD* = 5,4) characters per line.

### Experimental procedure

Goals were randomly presented to participants. For each goal, the presentation order of the six texts, along with their different relatedness to the goal, were set at random and participants did not know the distribution of the relatedness to the goal beforehand.

**Figure 2:**
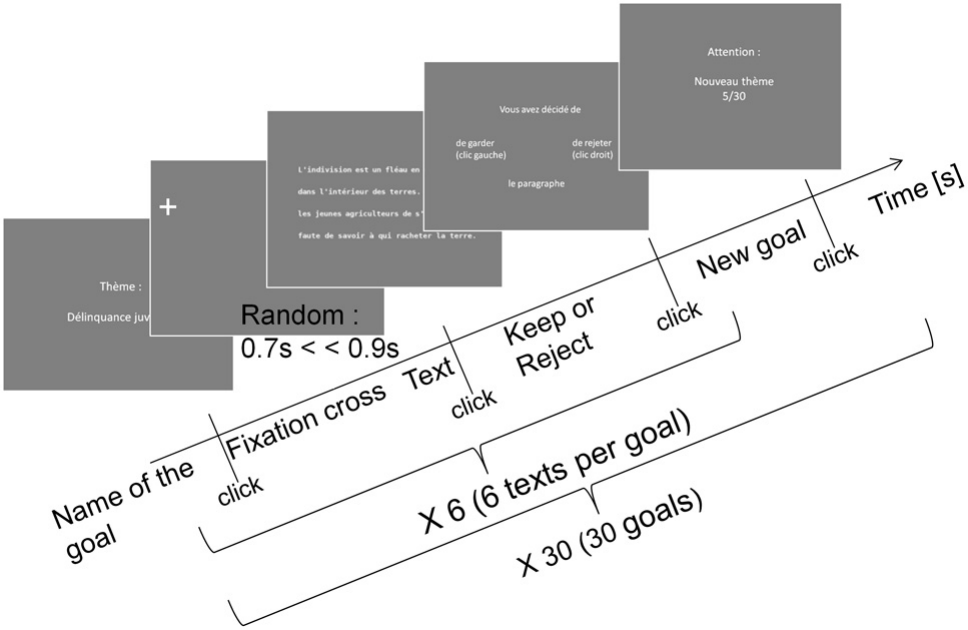
**Temporal sequence of a trial composed of different screens**.

The objective was to make a press review on given goals, by deciding as fast as possible whether the presented text had to be kept or rejected. Every participant made 180 decisions, i.e., 180 trials (30 goals × 6 texts). To ensure the correct understanding of the instructions, practice trials using one new goal and six texts were performed at the beginning of the experiment. Figure 2 describes the exact sequence of stimuli. A series of six trials began with the presentation of the goal, and then a fixation cross was displayed on the left of the first character of the first word for gaze stabilization. The duration of this period is random (mean = 800 ms, *SD* = 40 ms) to avoid the risk of saccade anticipation before the text presentation. The texts were displayed after this period of gaze stabilization. The mouse cursor remained invisible throughout the reading. Participants had to mouse-click as quickly as they could once they had taken a decision (to keep or reject the text). The next screen, with the visible mouse cursor, was then displayed to collect the participant's decision. Participants had to left-click on a green symbol to keep the text, or right-click on a red symbol to reject it. The trial was repeated six times for the same goal, and the whole procedure was repeated thirty times for the thirty different goals. In between goals, participants were given the opportunity to rest when necessary. They were also informed of the number of goals remaining until the end. A screen indicated that a new goal would be presented as well as the remaining goals count (Figure 2, last screen). The program describing the whole experiment was written in the Matlab environment, using Psychophysics Toolbox (Brainard, 1997) and SR Research's Eyelink library.

### EEG and eye tracking acquisition

Throughout the experiment, participants were comfortably seated in an adjustable chair while their EEG activity was being recorded. Thirty-one active electrodes (Brain Products GmbH) were mounted on an EEG cap (BrainCapTM) placed on their scalp in compliance with the International 10–20 system (Jasper, 1958). One electrode was affixed under the right eye to record vertical electro-oculographic activity (EOGv) with FP2 on the scalp. The information relating to the bipolar horizontal electro-oculographic (EOGh) signal was obtained through the eye tracking system. To get electrical contact and increase the signal-noise ratio, we have used contact gel (SuperVisc Gel, Brain Products, Inc.) and adjusted individual sensors until impedances were inferior to 5 kΩ.

Electrodes were referenced to head (FCz—ground:AFz), and EEG data were amplified through the BrainAmp™ system (Brain Products, Inc.), sampled at 1000 Hz, and then filtered with a 250 Hz low-pass filter.

For the sake of compatibility with this EEG acquisition, we have used the remote binocular infrared eye tracker EyeLink 1000 (SR Research) to track the gaze of each eye while the observer was looking at the screen. The EyeLink system was used in the Pupil-Corneal Reflection tracking mode sampling at 1000 Hz. For eye tracking acquisition purposes, the position of the head was stabilized with a chin rest and a fixed bar for the forehead. Participants were seated 68 cm in front of a 24-inch monitor (42 × 21° of visual field) with a screen resolution of 1024 by 768 pixels. The text was displayed at the centre of the screen (21 × 11° of visual field). While the text was displayed in average with 40.1 characters per line, each character covered 0.52° of horizontal visual angle, corresponding to about 3.8 characters in fovea.

At the beginning of the experiment, a 9-point calibration was operated. A drift correction was performed before every trial and a 9-point calibration would automatically be carried out again should the timeout on the initial fixation cross elapse or the experimenter decide to run it, in case an error above 0.5° was detected.

### Technical implementation

After acquisition, data (EEG raw data, hardware triggers, eye tracker raw data) had to be collected, synchronized and enriched with additional information on the read words, through a joint analysis of the scanpaths and associated texts (section Data enrichment: from fixations to words).

Raw data for both EEG (32 channels) and eye tracker (right and left eye positions) were sampled at 1 kHz. Besides, hardware triggers were automatically generated during the experiment to identify the different sequences (fixation cross, text/goal presentation …) in each trial. These marks were found both in the EEG file and the eye tracker file containing the raw data.

Even if both the EEG and eye tracker data were sampled at the same rate, data had to be synchronized to make up for clock drift, jitter and others sources of time distortions or information losses. This task was carried out through the known sequence of hardware triggers automatically generated during the experiment. After that, the raw data consisted in thirty-seven channels sampled at 1000 Hz: 32 for EEG data, 4 for eye tracker and one logical signal for the blinks detected by the eye tracker. After this global synchronization, eye tracker events (start, end of fixations and saccades) were added to the synchronized data file. The thresholds for saccade detection were a minimum velocity at 30°/ s, a minimum acceleration at 8000°/ s and a minimum motion at 0.1°/ s. These detections provided 12 eye tracker events (beginning / end of fixations, saccades and blink periods for both eyes).

### Data preprocessing

EEG data preprocessing and EFRP analyses were carried out with the Brain Analyzer 2 software (Brain Products GmbH, Version 2.0.2). Continuous EEG was first segmented over the whole duration of each text. A low cutoff filter was applied (2 Hz, Time constant 0.0796, 12 dB/oct.) as well as a notch filter at 50 Hz (symmetrical 5 Hz bandwidth around the notch frequency, i.e., 50 ± 2.5 Hz; 24 dB/oct.) to eliminate interference from the electricity network. Independent Component Analysis (ICA) was used to remove blink and saccadic movement artifacts from the EEG data (Makeig et al., 1997; Jung et al., 2000). Visual inspection of the FP2 channel, on which blinks are supposed to be maximal, in comparison with channels on which our effects were maximal (e.g., P4), showed that EOG artifacts were sufficiently removed from the data and that there was no significant impact from EOG artifacts on our results. Afterwards, epochs of 1200 ms were defined, starting 200 ms before the fixation onset. EFRPs analyses excluded the first fixations because they were related to the text onset (cf. Dimigen et al., 2011), as well as the last fixations that are known to be longer than intermediate position fixations (Just and Carpenter, 1980; Rayner et al., 2000) and to elicit specific EEG patterns (Hagoort, 2003). The baseline was defined between 200 and 100 ms before the onset of the fixation and then subtracted, because the artifact of the previous saccade was restricted from 100 to 0 ms before the fixation onset (Dimigen et al., 2011; Kamienkowski et al., 2012). Segments containing artifacts (bad gradients or excessive max-min) were rejected using a semi-automatic artifact rejection procedure. The Table 4 presented below (cf. Section Methodological aspects of EFRP analysis) shows, for each fixation of interest, the mean, standard deviation and minimum, across participants, of the remaining fixations after artifact rejection. Average EFRPs were then generated for every participant, electrode, and fixation of interest across trials. Among all available fixations, fixations of interest were selected based on semantic information taken from the different texts, as will be explained in the next section.

### Data enrichment: from fixations to words

Before data analysis, fixations whose duration was inferior to 80 ms or superior to 600 ms were excluded from the data (0.1% of all fixations). Fixation-related events were added *a posteriori* in order to characterize fixations according to both some semantic properties of the words read at each fixation and the fixation duration.

We had to predict which words were actually processed by participants for each fixation, in order to study EEG components that could have been induced by specific words. It is known that the area from which information can be extracted during a single fixation extends from about 3–4 characters to the left to 14–15 characters to the right of the fixation (Rayner, 1998). This area is asymmetric to the right and corresponds to the global perceptual span. Therefore, more than one word may be processed for a given fixation. It is hard to identify which words may have been processed for each fixation. As we mentioned earlier, staring at a word does not necessarily imply that it is processed. It could be processed when the eyes are still on the previous fixation, because it is highly predictable and/or partly distinguishable even from the parafoveal area. It could also be processed when the eyes are on the subsequent fixations, in case it was a low-frequency word whose processing spilled over onto the next word. EZ-Reader (Reichle et al., 1998, 2003) is a computational model that well describes this complex phenomenon by considering that eye movements and attention are decoupled, although processing is supposed to be performed one word at a time. It is therefore quite hard to guess exactly when each word is processed. Things get even worse as we fall within the SWIFT model (Engbert et al., 2005) because it assumes that a parallel processing could occur, i.e., several words could be processed at the same time. If there had been a consensus on a model, a solution could have been to run this model onto our data. Since it is not the case yet, we have ended up with the following method. We have used a window that was sized according to Rayner's assumptions. He has shown that the area from which a word can be identified extends no more than 4 characters to the left and 7–8 characters to the right of fixation, which corresponds to the word identification span. Moreover, Pollatsek et al. (1993) have shown that even if information taken from the next line was processed during a reading task, participants were not able of retrieving any semantic information. Therefore, the width of our window was 4 characters to the left plus 8 characters to the right of the fixation point. Since the initial fixations on the beginning of a word made it easier to recognize than initial fixations on the end of the word (Farid and Grainger, 1996), we have considered that a word is processed if at least the first third or last two-thirds of that word are inside the window.

Let us remember that we were interested in two particular decision-making situations:

#### Positive decision

This decision is made by participants once they find a semantic relationship between the goal and the current text. Our goal is to identify the premises of decision-making in the EEG signals, but we had to decide what part of the signals we had to look for, because the decision may occur some time before the mouse click. Several kinds of words were of interest as part of our investigation. Words with a high semantic relationship with the goal were choice words, but it would have been difficult to define a threshold for the relatedness to the goal. Words from the goal were also choice ones for two reasons: they were likely to be related to the goal and had been previously seen by the participant, which could also trigger the decision. For these reasons, we have selected those target words as potential markers of a positive decision. We have accepted all the words deriving from every word taken from the goal. For example, if the search goal was “croissance de l économie,” target words could be: “croissance,” “croissante,” “économie,” “économique,” “économiste,” etc.

#### Negative decision

This decision is made by participants once they are sure that there is no semantic relationship between the goal and the current text. Just like in the previous scenario, several words were of particular interest but we have ended up with incongruent words, i.e., words that have nothing to do with the goal and yet are specific enough of another domain. Therefore, their frequency is low and they are in no way related to the goal. We have empirically adjusted the thresholds to classify a word as incongruent, in order to meet two objectives. The first of these objectives was to have about the same number of target words read in highly related texts and of incongruent words read in unrelated texts. The second one concerned the participants' ability to effectively stop reading after they had read those words. For each participant, the number of remaining fixations after reading those words was computed in these two situations. Then, we adjusted thresholds to obtain similar distributions among participants. See section Results on eye tracker data on results of eye tracker data for more details. These two constraints were combined and the two thresholds, respectively set to 0.06 for the LSA semantic similarity, and 1.5 per million words for the word frequency. So, the fixated words, whose cosine with the goal was below 0.06 and word frequency below 1.5, were tagged as incongruent words.

**Figure 3:**
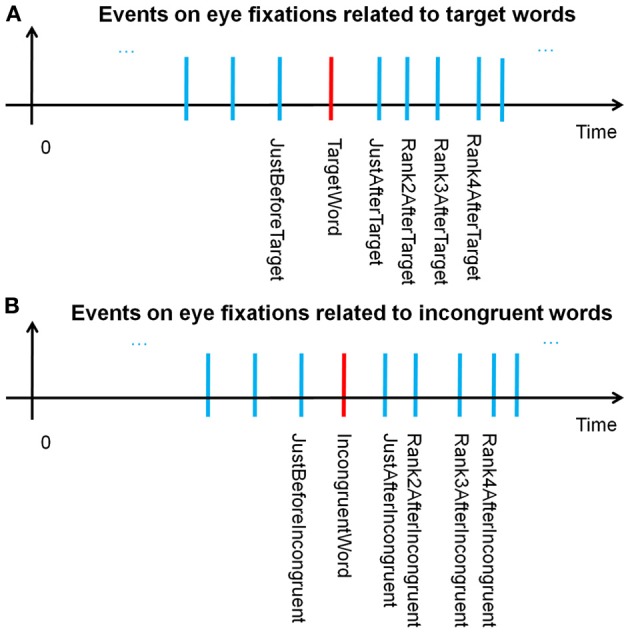
**(A)** Temporal sequence of events related to the fixations on, before and after the target word; **(B)** on, before, and after incongruent words.

Through the windowing mechanism, as explained above, both the fixations on incongruent words and target words were tagged after scanning the complete eye fixations dataset for all the subjects. These tagged fixations, labeled “Fixations of interest,” were specific events for EFRP analyzes, allowing the correct fixation selection before epoching. Figures 3A,B illustrates the temporal sequence of each and every event and provides a glossary of these event names. This glossary will be completed in section Fixation selection for global analysis. We will keep on referring to this glossary throughout the presentation of the methodology (sections Fixation selection for rank analysis and Fixation selection for global analysis), results and discussion.

### Statistics on words

We have computed some statistics about target and incongruent words and compared them to all other words.

The average length of target words is 7.8 characters (*SD* = 2.7) while the average length of incongruent words is 9.1 characters (*SD* = 3.4). Those words are slightly longer than all other words (5,4 characters, *SD* = 3.2). However, target words are about the same length as noun words (7.5 characters, *SD* = 2.6). Incongruent words are longer because they were selected from low frequency words and it is known that the length of a word tends to bear an inverse relationship to its relative frequency.

The average frequency of target words is 47.3 per million (*SD* = 52.4) while the average frequency of incongruent words is 0.4 per million (*SD* = 0.6). The frequency of target words lies in the central part of the distribution, whereas incongruent are by definition located in the left tail of the distribution.

## Results on eye tracker data

Out of the 17 participants, two were excluded from the EFRP analysis because of their behavioral data. One of them had read all the texts up to the end, so we considered that the decisions he made were not made during the reading process. Consequently, the average number of fixations for this participant was too high (over one standard deviation from the average of all participants). A second participant was excluded for the opposite reason; the average number of fixations for this participant was too low (under one standard deviation from the average of all participants). For the fifteen remaining participants, the average number of fixations and standard deviation for the three types of text (HR, MR, and UR) are indicated in Table 1.

**Table 1:** **Average number of fixations and standard deviation for all participants (15), according to the three kinds of text**.

**Text; Number of fixations**	**Mean**	***SD***
Highly related texts (HR)	18.1	7.8
Moderately related texts (MR)	23.2	8.6
Unrelated texts (UR)	17.2	7.7

HR texts are likely to induce positive decisions (keep the text), whereas UR texts should lead to negative decisions (reject the text). As regards the decision-making task performed by the participant, moderately related texts were created to introduce a continuum of relatedness to the goal, from unrelated to highly related, in order to maintain both the difficulty of the task and the necessity to read a significant part of the text before making any decision. In order to verify these assumptions, ratios for the kept and rejected texts were computed for each type of text. The ratio of correct responses (keep the text for highly related texts, and reject the text for unrelated texts) was very high (see Table 2, lines 2, 4, and 5). As regards moderately related texts, participants decided to keep the text in 47.2% of cases (about chance). These results confirm the role of neutrality for the MR texts in the experiment and their intermediate situation between UR and HR texts, which are our texts of interest.

**Table 2:** **Ratio of decisions for all the participants according to the different kinds of texts**.

**Text; Decision**	**Keep**	**Reject**
Highly related texts	836/900 (92.9%)	64/900 (7.1%)
Highly related texts with target words	188/207 (90.8%)	19/207 (9.2%)
Moderately related texts	425/900 (47.2%)	475/900 (52.8%)
Unrelated texts	45/900 (5%)	855/900 (95%)
Unrelated texts with incongruent words	11/210 (5.2%)	199/210 (94.8%)

Let us consider trials with target words and incongruent words, and also a temporal perspective on decision-making after those particular fixated words. Two issues were addressed. As we explained earlier, the first one was to set the selection thresholds for incongruent words. The second one was to analyze whether fixations on those words were linked to a possible speed-up in decision-making. In other words, do these specific words induce decision-making?

To empirically define the selection thresholds for incongruent words, two conditions were defined. On one hand, we wanted to have about the same number of trials with fixated target words and incongruent words. There were 32 target words in the 14 highly related texts, providing 207 trials where participants fixated target word(s) before making any decision. By setting the thresholds to 0.06 for the semantic link with the goal and 1.5 per million words for the word frequency, we have obtained 33 incongruent words used in 17 unrelated texts. We have observed 210 trials in which participants fixated incongruent words before making a decision. The number of trials with target words was about the same. On the other hand, we have aimed at an even distribution among participants of the median values of the number of remaining fixations after an incongruent word, but also after a target word. We have checked that these two patterns of median values were similar (minimum of mean squared error) as we changed the thresholds. Finally, the average for all participants of the median values of remaining fixations was 6.53 after a target word (*SD* = 2.20) and 5.76 (*SD* = 3.59) after an incongruent word. Then with the proposed thresholds, these two constraints were met.

The second calculation was intended to show that target and incongruent words are more likely to induce decision-making than other words. In trials with target words (Table 2, line 3), the percentage of correct response was high (90.8%) and not different from the overall trials on highly related texts (92.9%). In trials with incongruent words (Table 2, line 6), the percentage of correct response was high (94.8%) and not different from the overall trials on unrelated texts (95%). Then, fixation on a target or an incongruent word did not improve performance.

To analyze decision-making after a target word or respectively an incongruent word, we have compared the remaining number of fixations after those particular words (target or incongruent) and the remaining number of fixations after words which were equally fixed although they were NOT target or incongruent words. To do so, we have considered two couples of conditions: texts with fixations on target words (TW) vs. texts without fixations on target words (NTW), and respectively, texts with fixations on incongruent words (IW) vs. texts without fixations on incongruent words (NIW). In conditions TW and IW, for every participant and every text, we have computed the number of remaining fixations after target or incongruent words. For conditions NTW and NIW, for each participant all fixation ranks of target and incongruent words were randomly matched with texts without target words or incongruent words, in order to compute the remaining number of fixations. For example, suppose a participant made 12 fixations on a text, and fixated a target word with a rank of 9. The number of remaining fixations is 3. This case was randomly matched with another text without any target word. Suppose the reading of this text was abandoned after 14 fixations. The number of remaining fixations after 9 fixations is 5. This value of 5 will be compared with the value of 3 previously obtained. These matches were repeated 100 times.

In all the trials with target words, the average number of remaining fixations after a target word is 7.33. Using the same fixation ranks, this time for HR texts without target words, and considering 100 repetitions for matching, the average number of remaining fixations was 9.46 (*SE* = 0.14). In all the trials with incongruent words, the average number of remaining fixations after an incongruent word was 7.49. Now let us take the same fixation ranks, only this time on UR texts without incongruent words: considering 100 repetitions for matching, the average number of remaining fixations was 8.19 (*SE* = 0.11). In both cases, the remaining number of fixations after a target word or an incongruent word for a given rank was inferior to the remaining number of fixations after a word of the same rank that did not have those properties. These differences were highly significant (*p* < 0.01). From these behavioral data, we have concluded that fixations on target words or on incongruent words impacted decision-making since the effective decision to stop reading seemed to occur with a reduced latency from the fixation on those words, but did not impact the performance level.

Considering the fixation durations, the average and standard deviation of the fixation and saccade durations for both HR texts with target word(s) and UR texts with incongruent word(s) are indicated in Table 3. The fixations with a duration inferior to 80 ms or superior to 600 ms were excluded from analyses (0.1% of the whole fixations).

**Table 3:** **Average number of fixations, average and standard deviation for fixation durations and saccade durations for all the participants according to the types of text**.

	**Fixation**	**Fixation duration**		**Saccade duration**	
	**Number**	**Mean [ms]**	***SD* [ms]**	**Mean [ms]**	***SD* [ms]**
HR texts with target words (15 §)	3965	184.5	63.7	45.8	30.6
UR texts with incon-gruent words (17 §)	4121	182.0	64.4	45.7	30.3

**Figure 4:**
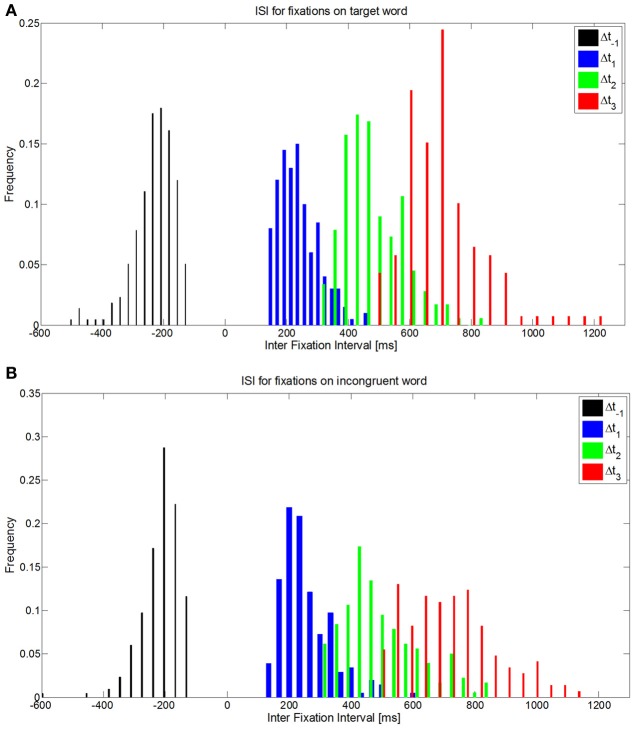
**Distribution of ISI Δ*t*_-1_, Δ*t*_1_, Δ*t*_-2_ when fixations of interest are fixations on (A) target words, or (B) on incongruent words**.

To carry out EFRP analyses, it is important to know the distribution of the intervals between fixations, which constitute the inter stimuli interval (ISI) for ERP extraction. The duration of the inter stimuli interval corresponds to the sum of the durations of one fixation and one saccade (Table 3). Then considering these statistics, we observe a continuum of events coming from previous and subsequent fixations, for a given time interval in EFRP analysis. To illustrate this, let us note Δ*t*_1_, respectively Δ*t*_2_, the temporal interval between a given fixation of interest and the first subsequent, respectively the second subsequent, and also Δ*t*_−1_, the temporal interval with the first previous fixation. Such distributions on Δ*t*_−1_, Δ*t*_1_, and Δ*t*_2_ are plotted in Figure 4, where the fixations of interest are all the fixations on target words (Figure 4A), or all the fixations on incongruent words (Figure 4B), for all the subjects. We will discuss these statistical properties of ISI in EFRP when interpreting the results of our EFRP analyses (section Discussion, Conclusion). Consequently for EFRP, if we consider a time interval of around 300 ms to extract a component, the resulting signal pattern will reflect the convolution of the EEG activity elicited at the onset of both the fixation and the subsequent fixation, since in average, the duration of one fixation cumulated with one saccade –185 + 45 ms = 230 ms- is inferior to 300 ms. The same argument applies to a time interval of around 500 ms, the result of EFRP analysis will reflect the convolution of the EEG activity elicited at the onset of both the fixation and the two subsequent fixations, while in average the duration of two fixations cumulated with two saccades −2 × (185 + 45 ms) = 460 ms- is inferior to 500 ms. We will discuss these situations in sections EFRP analysis for late components and results and discussion, conclusion.

## Methodological aspects of EFRP analysis

In this study, our objective was to analyze the neural activities of decision-making during the reading of a text on the time scale of the eye fixations. The methodology for EFRP analyses therefore combined both the selection of particular fixations in the texts and the temporal evolution of neural activities, before and after these events. Besides, because of brain signal overlaps resulting from a much smaller average inter stimuli interval (230 ms including the durations of the fixation and the saccade) than the usual latency of late components, EFRP were analyzed according to a three-step strategy.

Firstly, EFRP were studied at the level of fixation ranks. Brain signals were averaged for each fixation events preceding or following a fixation on a target word or an incongruent word. More precisely, we first averaged brain signals at the onset of fixations on the target word. Then, we averaged brain signals on fixations occurring just before the target word, but also on fixations Target−2, Target−3, etc. We did the same on fixations following the target word: Target+1, Target+2, etc. We have used the same principle for the incongruent words. Rank analyses were analyzed through temporally linked conditions, in order to study the time course of neural activities, fixation after fixation, around particular events. The goal was to look for fixation ranks that could be associated with a specific brain component that would be a marker of a decision.

Secondly, we focused on those fixation ranks showing a specific brain signal pattern that was different from the other surrounding ranks, in order to carry out a more global analysis. To study whether a particular signal pattern we had found was really specific to a given rank, we have compared it to every signal that appeared both at previous fixation ranks and subsequent ones. Actually, we have made a selection of those events so as to guarantee an even distribution of fixation duration before and after the central event.

Finally, we investigated late components synchronized with these particular events, in spite of the fact that after about 230 ms, the signal overlaps with the one associated with the *N* + 1 fixation and, even worse, at about twice that time, the signal overlaps with that of the *N* + 2 fixation. For these analyses, the expected latencies will be deduced from the previous results. The Table 4 shows the mean, standard deviation and minimum for all the fixations of interest as mentioned earlier.

**Table 4:** **Mean, Standard deviation (*SD*), and Minimum (Min) across participants for all fixations of interest, for target and incongruent words, after preprocessing**.

	**JustBefore target**	**Target**	**JustAfter target**	**Rank2After target**	**Rank3After target**	**Select beforeT**.	**Select afterT**.	
Mean	12.9	13.8	12.1	11.3	8.6	43.7	40.6	
*SD*	2.1	2.4	2.1	2.1	1.5	14	16.7	
Min	9	9	7	6	6	27	19	
	**JustBefore Inc**.	**Inc**	**JustAfter Inc**.	**Rank2After Inc**.	**Rank3After Inc**.	**Rank4After Inc**.	**Select beforeI**	**Select afterI**
Mean	13.3	14	13.2	11.9	9.9	8.9	52	21.8
*SD*	3.7	3.8	4.3	4.1	3.5	3.8	31.6	11.6
Min	8	8	6	5	5	3	16	6

**Figure 5:**
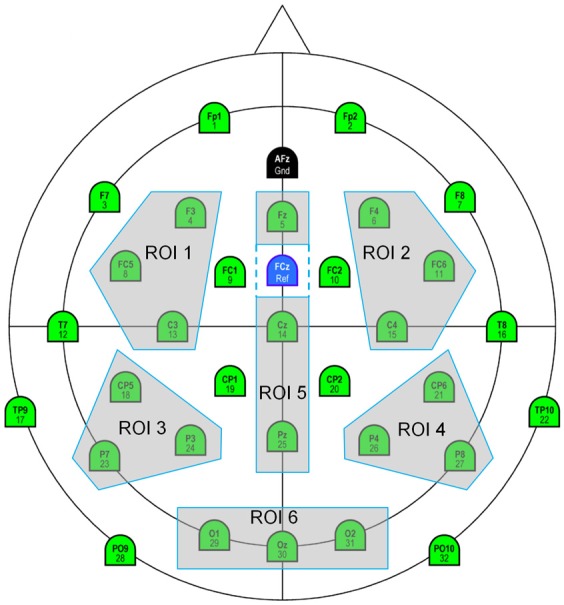
**Six Regions of Interest selected: ROI 1 (fronto-central left): F3, FC5, C3; ROI 2 (fronto-central right): F4, FC6, C4; ROI 3 (centro-parietal left): CP5, P7, P3; ROI 4 (centro-parietal right): CP6, P4, P8; ROI 5 (midline): Fz, Cz, Pz; ROI 6 (occipital): 01, 0z, 02**.

For all these analyses and for each participant, a voltage average has been computed on different latency windows, for all fixations of interest and Regions of Interest, (ROI, see Figure 5), each grouping three electrodes and were subjected to ANOVAs. ROI 1, 2, 3 and 4 were selected to allow left (ROI 1, ROI 3) vs. right (ROI 2, ROI 4) and anterior (ROI1, ROI2) vs. posterior (ROI 3, ROI 4) comparisons. ROI 5 includes midline electrodes (Fz, Cz, Pz) excepted Oz, because we wanted, due to our task, to put together all the occipital electrodes (ROI 6: O1, Oz, O2). *P*-values were reported after the Greenhouse-Geisser correction for nonsphericity and Tukey tests were used for *post-hoc* comparisons.

## Rank analysis and results

### Fixation selection for rank analysis

According to our assumptions, the aim of the fixation-by-fixation EFRP analysis, called *rank analysis*, was to search for EEG components which would be related to decision-making consecutively to fixations on target words or incongruent words. Figure 3A illustrates the temporal position of all the events related to the fixations on, before and after target words. For instance, the fixation-related event that is situated two fixations after the fixation on the target word is called “Rank2AfterTarget.” Figure 3B illustrates the temporal position of all the events related to the fixations on, before and after incongruent words. Thanks to all these events, we first conducted different EFRP analyzes, rank by rank, centered on “TargetWord” and “IncongruentWord” events. All these *rank analyses* are presented in section Results of rank analysis on target words concerning the events related to target words, and in section Results of rank analysis on incongruent words concerning the events related to incongruent words.

These rank analyzes thus allowed us to detect EEG components which could potentially be related to decision-making, but also to characterize these neural patterns as a transient response. The result of these *rank analyses* was the selection of key events on which a more *global analysis* was carried out to characterize the elicited EEG components with more accuracy.

### Results of rank analysis on target words

After the inspection of the EFRPs data and review of the relevant literature (Key et al., 2005; Polich, 2010), we selected respectively the 0–50, 51–90, and 91–200 ms latency windows for analysis. Indeed, as seen in the following results, an effect was observed in the first positive component. According to us and regarding to our task, this early effect was interpreted as a late effect of previous fixations (cf. Section EFRP analysis for late components and results), specifically in this case as an effect on the P300 component. The Fixations of interest included in the ANOVAs were: JustBeforeTarget, TargetWord, JustAfterTarget, Rank2AfterTarget and Rank3AfterTarget.

#### 0–50 ms latency window

No significant effect was found.

#### 51–90 ms latency window

The Fixations by ROI interaction was significant between JustBeforeTarget, JustAfterTarget and Rank2AfterTarget fixations [*F*_(10, 140)_ = 2.27, *p* = 0.05]. At ROI 4, JustAfterTarget events elicited a larger positivity than both JustBeforeTarget (*p* = 0.0001) and Rank2AfterTarget events (*p* = 0.01). At ROI 6, JustAfterTarget events elicited a larger positivity than JustBeforeTarget ones (*p* = 0.043).

**Figure 6:**
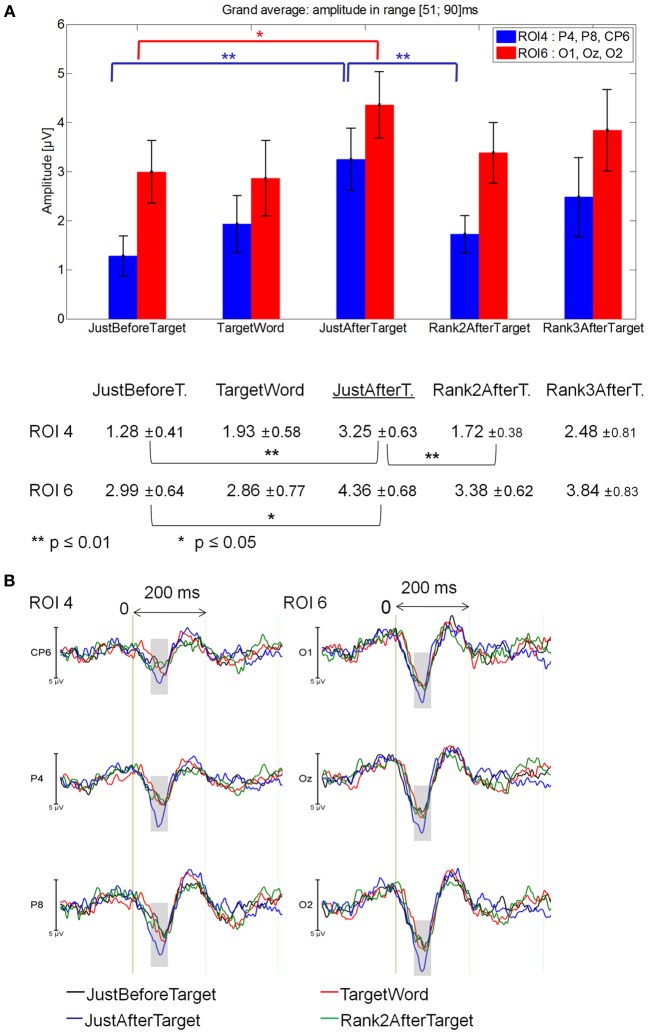
**Grand average EFRP for rank analyzes, at ROI 4 and ROI 6 for fixations on, just before, just after and two fixations after target words: (A) mean amplitudes and standard errors (μV) in the 51–90 ms latency window, where differences were significant**. Key event was underlined; **(B)** EFRP on four consecutive events. The amplitude of the effects is represented on the ordinate (in μV; negativity is up). The time is on the abscissa (in ms), 0 is the onset of each Fixation of interest. The gray areas indicate the latency window in which differences were significant.

No significant difference was found between Target and JustAfterTarget fixations or between Target and JustBeforeTarget fixations [*F*_(2, 28)_ = 1.48, *p* = 0.24]. Likewise, no significant difference was found between Rank2AfterTarget and Rank3AfterTarget fixations [*F*_(1, 14)_ = 0.30, *p* = 0.59]. See Figure 6A for mean values and standard errors.

#### 91–200 ms latency window

No significant effect was found.

These results (Figure 6B) showed that the first positive component observed after the onset of each fixation was larger for the fixation just after that on the target word. This effect was located in the right centro-parietal and occipital areas. We will call this JustAfterTarget event *key event* throughout the rest of the paper.

### Results of rank analysis on incongruent words

We have selected the 0–50, 51–120 and 121–200 ms latency windows for the analysis, after visual inspection of the traces and regarding to the literature (Camblin et al., 2007; Kutas and Federmeier, 2011). Indeed, data inspection showed an early negative effect that was interpreted as an N400 modulation of previous fixations (cf. Section EFRP analysis for late components and results). The fixations of interest included in the ANOVAs were: JustBeforeIncongruent, IncongruentWord, JustAfterIncongruent, Rank2AfterIncongruent, Rank3AfterIncongruent, and Rank4AfterIncongruent.

#### 0–50 ms latency window

No significant effect was found.

#### 51–120 ms latency window

Rank2AfterIncongruent (−0.28μV) elicited a lower positivity than for JustBeforeIncongruent events (0.81 μV; main effect of Fixation: [*F*_(1, 14)_ = 7.12, *p* = 0.018], specifically at ROI 3 (*p* = 0.0018) and ROI 6 (*p* = 0.00012; Fixation by ROI interaction: [*F*_(5, 70)_ = 2.90; *p* = .043].

Rank2AfterIncongruent event also elicited a lower positivity than for IncongruentWord event [0.82 μV; main effect of Fixation: *F*_(1, 14)_ = 4.25, *p* = 0.05].

**Figure 7:**
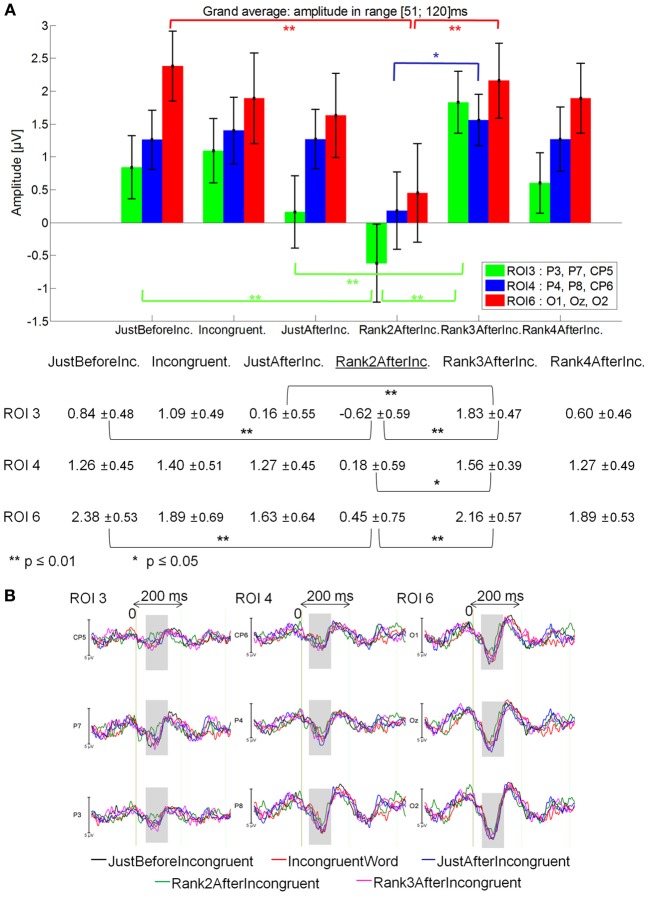
**Grand average EFRP for rank analyzes, at ROI 3, ROI 4, and ROI 6, for the fixations on and around incongruent words: (A) mean amplitudes and standard errors (μV) in the 51–120 ms latency window, where differences were significant**. Key event was underlined; **(B)** EFRP on five consecutive events. The amplitude of the effects is represented on the ordinate (in μV; negativity is up). The time is on the abscissa (in ms), 0 is the onset of each Fixation of interest. The gray zones indicate the latency window in which differences were significant.

Finally, Rank2AfterIncongruent fixations were less positive than Rank3AfterIncongruent fixations [1.16 μV; *p* < 0.05; main effect of Fixation: *F*_(2, 28)_ = 3.39; *p* = 0.05], specifically in the ROI 3, ROI 4 and ROI 6 Fixation by ROI interaction marginally significant [*F*_(10, 140)_ = 2.25; *p* = 0.07], while no significant difference was observed with JustAfterIncongruent fixations. See Figure 7A for mean amplitude and standard error values.

No significant difference was observed between JustBeforeIncongruent, Incongruent and JustAfterIncongruent fixations [*F*_(2, 28)_ = 0.086, *p* = 0.87], or between Rank3AfterIncongruent and AfterRank4Incongruent [*F*_(1, 14)_ = 0.83, *p* = 0.38; Fixation by ROI interaction: *F*_(5, 70)_ = 1.28, *p* = 0.29].

#### 121–200 ms latency window

No significant effect was found.

These results showed (Figure 7B) that the first positive component elicited by the second fixation after that on the incongruent word was less positive. This effect is specifically located in centro-parietal and occipital areas. This Rank2AfterIncongruent event will be called *key event* throughout the rest of the paper (underlined in Figure 7).

## Global analysis and results

### Fixation selection for global analysis

The objective of the global analysis was to observe the EEG components in comparison with after the previous and next ones, considering more than one specific fixation, contrary to the rank analysis. In order to clarify things, let us explain the global analysis, taking the example of fixations on target words. The principle is the same for the incongruent words and we will notice only the specific differences between these two cases.

Among other things, the rank analysis on target words (section Results of rank analysis on target words) allowed us to detect a neural cue on the fixation just after the target words (JustAfterTarget event). In this case, this event is our *key event*. For the incongruent words, the *key event* is the Rank2AfterIncongruent one (section Results of rank analysis on incongruent words).

**Figure 8:**
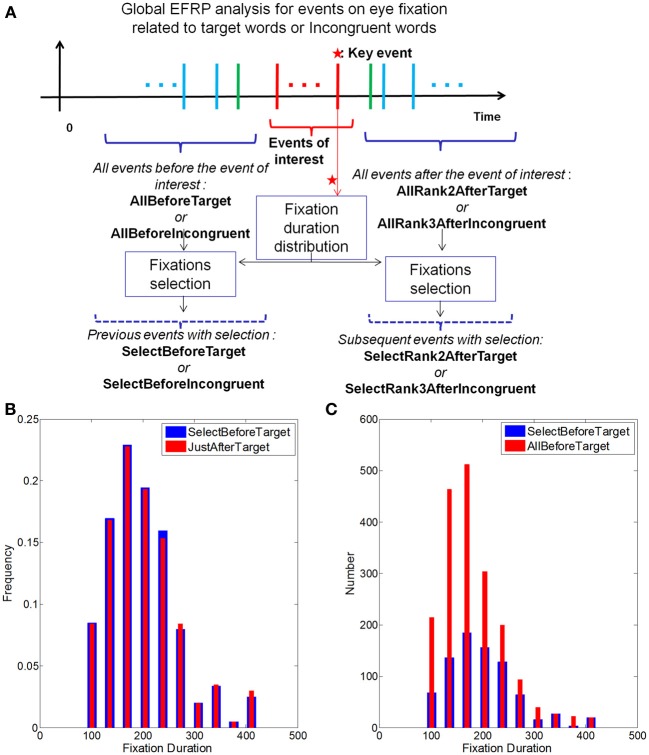
**(A)** Temporal sequence of the collected events and selection of the fixations related to the previous and subsequent events having the same distribution of fixation duration as that of the key event (represented by stars). **(B)** Result of the fixations selection related to the events before “TargetWord” in order to have the same distribution of fixation durations as the key event (“JustAfterTarget”); **(C)** In each bin of the histogram for the “BeforeTarget” event, random selection of the fixations for the matching distribution. The result is the histogram (“SelectBeforeTarget”) after selection **(B)**.

Based on these key events, we have defined more global events, gathering previous and subsequent events. Subsequent events consisted of the fixations after the key event (i.e., for the target words: Rank2AfterTarget, Rank3AfterTarget …) until the penultimate fixation before the participant decides to stop reading. Likewise, previous fixations included the fixations before the target word: from the JustBeforeTarget fixation up to the second one (the first fixation at the onset of the text presentation was excluded). Finally, in order to minimize the effect of the fixation duration variability between the three previously defined populations, namely key event (i.e., Rank2AfterTarget or Rank3AfterIncongruent), before key events (i.e., AllBeforeTarget or AllBeforeIncongruent) and after key events (i.e., AllRank2AfterTarget or AllRank3AfterInconguent), we have made a selection of fixations (Nikolaev et al., 2011), so that fixations before the key event and fixations after the key event followed the same distribution of durations as the key event (see Figure 8).

To do so, the distribution of fixation durations for the key event has been computed (in red, in Figure 8B), as well as histograms of fixation durations for the surrounding events (in red, in Figure 8C). For each bin, the number of selected fixations is computed to be proportional to the distribution on the key event and select a maximum number of fixations (in blue, in Figure 8C). This selection was made according to a uniform random sampling. The selected set of fixations composed a new event, called SelectBeforeTarget event. The same procedure has been applied to generate the set of fixations related to the SelectRank2AfterTarget event. This method has been replicated for the selection of fixations related to the previous and subsequent events in the case of incongruent words (SelectBeforeIncongruent and SelectRank3AfterIncongruent to match the distribution of fixation durations on the key event Rank2AfterIncongruent).

The global analysis focused on the contrast between the key event and both the selected previous events and selected subsequent events.

### Results of the global analysis on target words

The selected latency windows were the same as those of the rank analysis (0–50, 51–90, and 91–200 ms). The fixations of interest included in the ANOVAs were: SelectBeforeTarget, JustAfterTarget and SelectRank2AfterTarget.

#### 0–50 ms latency window

No significant effect was found.

#### 51–90 ms latency window

**Figure 9:**
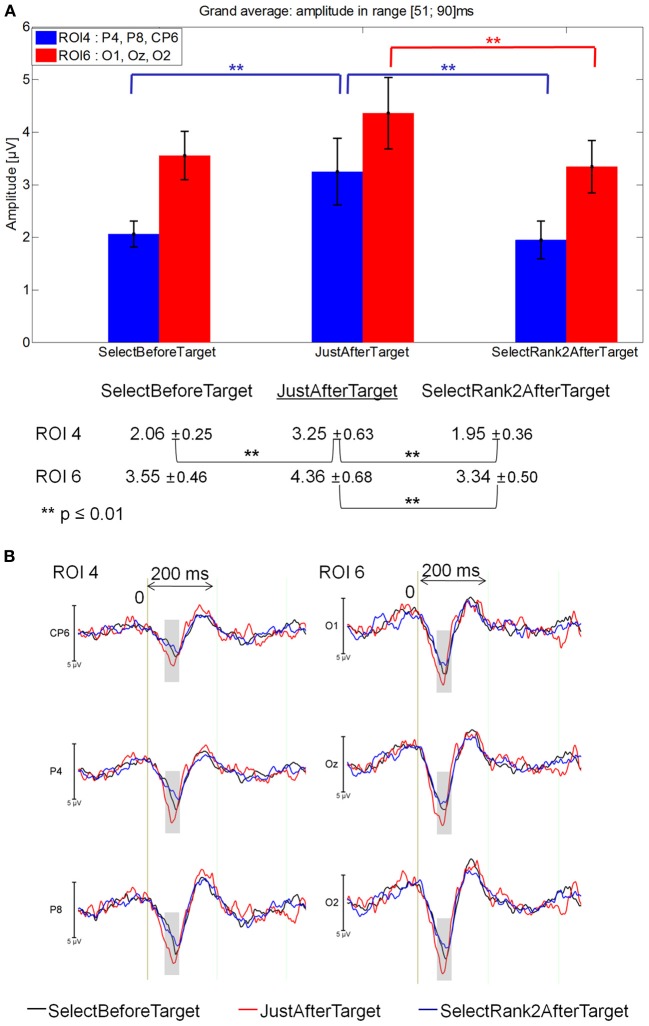
**Grand average EFRP for global analyzes, at ROI 4 and ROI 6, for fixations just after, selected before and selected after target words: (A) mean amplitudes and standard errors (μV) in the 51–90 ms latency window, where differences were significant**. Key event was underlined; **(B)** EFRP on selected fixations. The amplitude of the effects is represented on the ordinate (in μV; negativity is up). The time is on the abscissa (in ms), 0 is the onset of each Fixation of interest. The gray zones indicate the latency window in which differences were significant.

The Fixation by ROI interaction was significant between SelectBeforeTarget, JustAfterTarget and SelectRank2AfterTarget [*F*_(10, 140)_ = 2.83, *p* = 0.026). At ROI 4, JustAfterTarget elicited a larger positivity than for both SelectBeforeTarget and SelectRank2AfterTarget fixations (*p* = .00041 and.00007, respectively) that did not differ from each other. At ROI 6, JustAfterTarget event elicited a larger positivity than for SelectRank2AfterTarget event (*p* = 0.0065). See Figure 9A for mean amplitude and standard error values.

#### 91–200 ms latency window

No significant effect was found.

These results showed that the first positive component elicited by the fixation just after that on the target word (i.e., JustAfterTarget) was more positive than both the selected previous and subsequent fixations (i.e., SelectBeforeTarget and SelectRank2AfterTarget) in the right centro-parietal areas and the selected subsequent fixations (i.e., SelectRank2AfterTarget) in the occipital areas (Figure 9B).

### Results of the global analysis on incongruent words

The selected latency windows were the same as those of the rank analysis (0–50, 51–120, and 121–200 ms). The fixations of interest included in the ANOVAs were: SelectBeforeIncongruent, Rank2AfterIncongruent and SelectRank3AfterIncongruent.

#### 0–50 ms latency window

No significant effect was found.

#### 51–120 ms latency window

**Figure 10:**
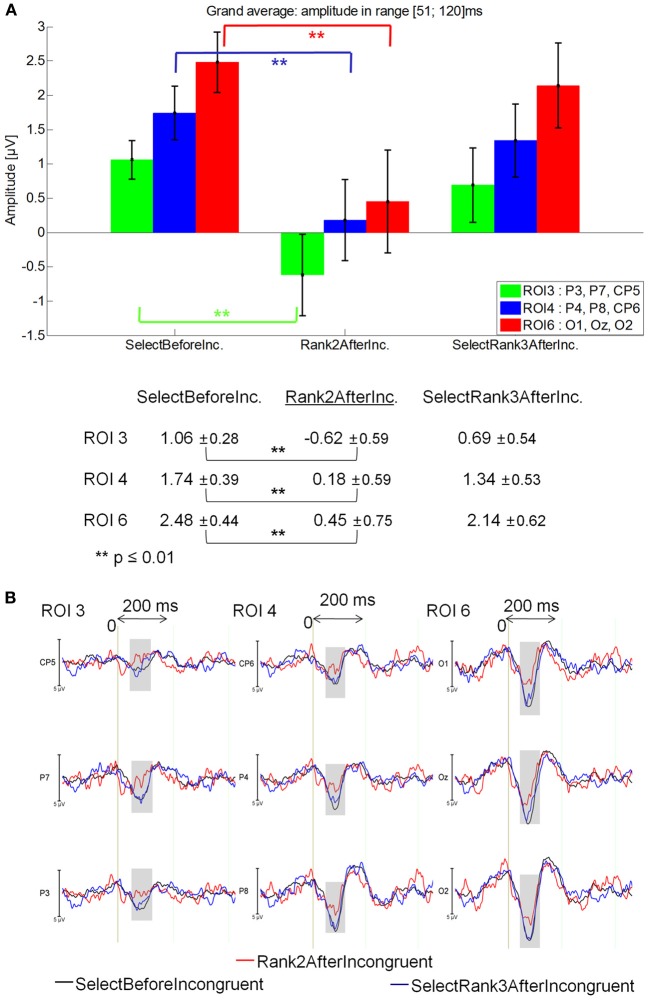
**Grand average EFRP for global analyzes, at ROI3, ROI 4, and ROI 6, for fixations on and selected fixations around incongruent words: (A) mean amplitudes and standard errors (μV) in the 51–120 ms latency window, where differences were significant**. Key event was underlined; **(B)** EFRP on selected fixations. The amplitude of the effects is represented on the ordinate (in μV; negativity is up). The time is on the abscissa (in ms), 0 is the onset of each Fixation of interest. The gray zones indicate the latency window in which differences were significant.

Rank2AfterIncongruent was less positive (−0.28μV) than in SelectBeforeIncongruent (1.05 μV) and SelectRank2AfterIncongruent [0.80 μV; main effect of Fixation: *F*_(2, 28)_ = 4.38, *p* = 0.023], which did not differ from one another. Specifically, Rank2AfterIncongruent was less positive than SelectBeforeIncongruent at ROI 3 (*p* = 0.00013), ROI 4 (*p* = 0.00017) and ROI 6 *p* = 0.00012; Fixation by ROI interaction: [*F*_(5,70)_ = 2.89, *p* = 0.049]. See Figure 10A for mean amplitude and standard error values.

#### 121–200 ms latency window

No significant effect was found.

These results show that the first positive component elicited by the Rank2AfterIncongruent fixations was less positive than selected previous fixations (i.e., SelectBeforeIncongruent) in centro-parietal and occipital areas (Figure 10B).

## EFRP analysis for late components and results

### EFRP analysis for late components

The previous analyses revealed that there is a more negative component in the right centro-parietal and occipital areas on fixation N+2 after the incongruent word than on the previous fixations. The negativity occurred quite early, starting 50 ms after the onset of the incongruent word. Since a negative decision made on an incongruent word is likely to be based on a complex semantic process, we suspected that this negativity could be due to a late component of the incongruent word processing. This component would be strong enough to remain visible after it is merged with components from the two subsequent fixations.

The same argument applies to the target words. We found a specific higher positivity right after the onset of the fixation following the target word. It could also be due to a late component of the target word processing.

As we mentioned earlier, the mean fixation duration is about 185 ms and the saccade duration about 45 ms. So there are about 230 ms from fixation N to N+1 and 460 ms from fixation N to N+2. Since we found a specific component that started around 50 ms after the beginning of the fixation on the Target+1 event as well as on the Incongruent+2 event, we have looked for late components after these durations, that is after 230 + 50 ms for fixations on target words and after 460 + 50 ms for fixations on incongruent words.

Late component were analyzed respectively in the 260–320 ms latency window for target words and in the 500–530 ms latency window for incongruent words. Those latency windows were chosen after visual inspection of EFRPs data, review of the relevant literature (Key et al., 2005; Polich, 2010 for target words analysis; Camblin et al., 2007; Kutas and Federmeier, 2011 for incongruent words analysis) and, for their beginning, calculation of average fixation duration (cf. above). The fixations of interests were: JustBeforeTarget, TargetWord and JustAfterTarget for target word analysis, and JustBeforeIncongruent, IncongruentWord and JustAfterIncongruent for incongruent word analysis.

### Late components on target words

**Figure 11:**
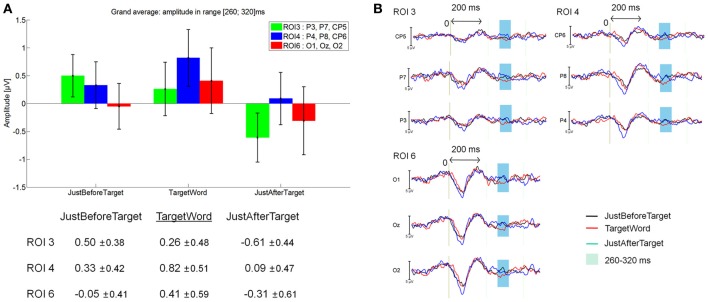
**Grand average EFRP for analyses of late components, at ROI3, ROI 4, and ROI 6, for the TargetWord event compared to the previous and subsequent ones: (A) mean amplitudes and standard errors (μV) in the 260–320 ms latency window; (B) EFRP on three consecutive fixations**. The amplitude of the effects is represented on the ordinate (in μV; negativity is up). The time is on the abscissa (in ms), 0 is the onset of each Fixation of interest.

As shown on Figure 11B, a late positive component seemed to be arising on the 260–320 latency window, which was linked to the fixations on target words (red line). However, no significant effect was found between JustBeforeTarget (0.14 μV), TargetWord (0.36 μV) and JustAfterTarget (–0.17 μV; *F*_(2, 28)_ = 0.74, *p* = 0.48 for the main effect of Fixation, and *F*_(10, 140)_ = 0.94, *p* = 0.45 for the Fixation by ROI interaction) in this latency window. See Figure 11A for mean amplitude and standard error values for ROI 3, 4, and 6. This late positivity elicited by target words could be interpreted as a P300 component.

### Late components on incongruent words

#### In the 500–530 ms latency window

**Figure 12:**
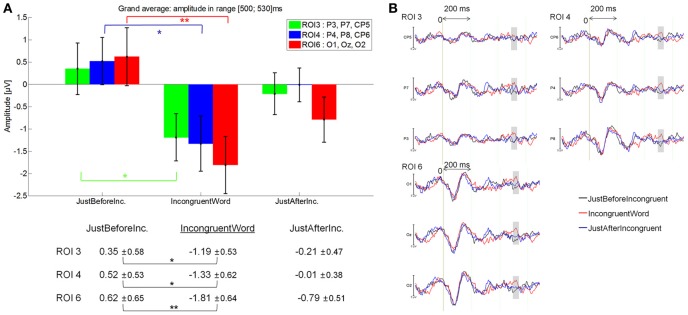
**Grand average EFRP for analyses of late components, at ROI3, ROI 4 and ROI 6, for the IncongruentWord event compared to the previous and subsequent ones: (A) mean amplitudes and standard errors (μV) in the 500–530 ms latency window, where differences were significant; (B) EFRP on three consecutive fixations**. The amplitude of the effects is represented on the ordinate (in μV; negativity is up). The time is on the abscissa (in ms), 0 is the onset of each Fixation of interest. The gray zones indicate the latency window in which differences were significant.

IncongruentWord elicited a larger negativity (−0.93 μV) than JustBeforeIncongruentWord [0.37 μV; main effect of Fixation: *F*_(2, 28)_ = 3.17, *p* = 0.05], specifically at ROI 3 (*p* = 0.042), ROI 4 (*p* = 0.005) and ROI 6 (*p* = 0.00016); Fixation by ROI interaction: [*F*_(5, 70)_ = 3.13, *p* = 0.043]. See Figure 12A for mean amplitude and standard error values. No significant difference was observed between IncongruentWord and JustAfterIncongruentWord (−0.063 μV, *p* = 0.24).

These results showed that fixations on incongruent words elicited a larger negativity than fixations just before incongruent words in centro-parietal and occipital areas (Figure 12B). The latency window (500–530 ms) and scalp distribution of this negative component suggest that we identify it as an N400 component.

## Discussion, conclusion

Decision-making is a fundamental activity of the search for information in texts. The aim of our study was to highlight specific EFRPs patterns linked to decision-making, in a task where participants had to decide as quickly as possible whether the text currently read was semantically related to a given goal.

The task we have designed is much closer to the natural search for information than what literature usually describes. Reading is often temporally or spatially constrained in order to facilitate the alignment of EEG signals: words are studied separately or slowly presented one at a time. In this particular case, words are displayed all at once on the screen, covering multiple lines, exactly like the texts we are used to read every day.

The downside of this ecological approach is that signal patterns are much harder to analyze because of overlapping processes. The reason is that as early as 230 ms (a fixation of about 185 ms in average plus a saccade of about 45 ms) after the onset of a fixation, a new fixation occurs which produces a new signal pattern scrambling the previous one.

To reduce that noise, we could have tried to control the material very precisely. Actually, we controlled the overall relatedness of the text to the goal, but it was not easy to do so with all the factors that affect reading in a full text and which would have facilitated the analyses: word frequency, word predictability, word-goal relatedness distribution over the text. Therefore, we have picked up a high number of real texts, more or less associated to a large variety of goals. The idea was to have a large text variability in order to expect a compensation of brain signal peculiarity. For instance, suppose we are interested in the brain waves induced by fixations F. There are two ways to solve the issue of the overlapping F and F+1 fixations. The first one is to guarantee that all F+1 fixations elicit the same signal, which could then be easily subtracted from the main signal (Woldorff, 1993). This is almost impossible to do with a textual material, because of the high number of factors involved. The second way is therefore to select a large variety of texts in order to expect that F+1 fixations elicit signals that would counterbalance each other and as a whole would not affect the main signal too much, considering that temporal jitters on previous and subsequent events act as high frequencies filters. The higher the fixation, the larger the jitter. Then, the overlapping effect can be reduced for late components.

### The proposed methodology for EFRP analysis

**Figure 13:**
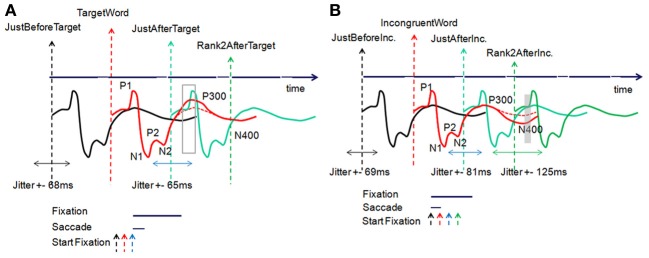
**Timeline of the EFRP elicited at each fixation, (A) for target word processing, (B) for incongruent word processing**. See text for explanations of the different signals.

To overcome this difficulty, sophisticated methods for uncovering specific EEG patterns during reading and before making the final decision were therefore initiated. The task is complex since at least two different processes are intertwined (reading and decision-making) and this intertwining depends on both the structure of the text and the participant. Consequently, rank analyses, either forward from the first fixation or backward from the last one, are inappropriate. Moreover, extractions of EFRP from words that have particular properties (frequency, predictability,…) independently of the fixation rank (Kliegl et al., 2012) is not our purpose, while in this case we have explicitly asked the participants to make decisions as they were reading. Our methodology is therefore a hybrid of these two cases. We first identified choice words likely to elicit specific components. We then performed an analysis at the level of fixations, around these words, without any assumption on the latency. By resorting to the EFRPs technique, which allows for a fixation-by-fixation analysis of the EEG signal, we identified two response patterns, related respectively to the first fixation just after a target word and the second one after an incongruent word: the fixation just after a target word elicited a larger first positive component in the right parieto-occipital areas and the second fixation after an incongruent word elicited a lower first positive component in the parieto-occipital areas (bilateral scalp distribution). These effects were very early (50–90 and 50–120 ms after the fixation onset) and can hardly be interpreted as the reflection of a decision made in relation to a semantic process. This is why we have mixed EFRP analyses at two different scales: at the scale of one ISI duration to extract early components, and at the scale of 2 or 3 times the ISI duration to extract late components. In the first case, the early extracted components cannot be interpreted as the reflection of a semantic process beginning at the onset of the current fixation. However, they reveal the overlapping of current and previous activities started one or two fixations earlier. In the second case, the late components observed with a latency about 2 or 3 times the ISI duration are also corrupt. In some situations, thanks to the natural ISI jitter, these late components can be observed (depending on the relation between the jitter range and the temporal frequency band of the component). Then, while in these two cases the extracted components are corrupt, the results of these two analyses must be congruent to allow for an interpretation of the extracted component (Figures 13A,B). Let us consider typical ERP components in the centro-parietal areas, which are related to word processing during reading (Sereno et al., 1998). Such a signal elicited at each fixation is illustrated in Figures 13A,B. Then, our hypothesis is that late components, such as P300 or N400, could be superimposed on one or two additional components corresponding to the first and second subsequent fixations.

### Positive decision (target) indexed by a P3b?

We have defined target words as words that must have the two following characteristics: on the one hand, a morphological form that corresponds to the goal of the text, and on the other hand, a high semantic association with the goal. In the light of these two preceding criteria, we had hypothesized that these words would be likely to induce a (positive) decision to leave the text. Our behavioral data support this hypothesis and show that the remaining number of fixations after a fixation on a target word was significantly reduced when compared with the remaining number of fixations after any other word that was not a target word while being at the same rank of fixations. Thus, decision-making involves accumulation of information, and in our case, with the same preceding amount of information, target words apparently provide a large enough gap to induce a decision.

In terms of electrophysiological data, these two characteristics of the target words (i.e., same verbal form as the goal and high semantic link with the goal) may result in different components. As regards the morphological form, a consensus seems to emerge in the literature showing early electrophysiological effects related to its processing. The first one, the P150, is a relatively focal positive component elicited in the occipital sites (especially in the right hemisphere), which is more positive when a target word is not related to a prime word, in comparison with a full or partial (one letter changed) repetition (Holcomb and Grainger, 2006). This component may not be language-specific and has also been observed in experiments with single letters (Petit et al., 2006) and pictures of objects (Eddy et al., 2006), with the same latency and scalp distribution, suggesting that it would reflect an early process related to surface features and the mapping of visual features onto higher level representations (Chauncey et al., 2008). Note that the latency of P150 overlaps that of N1, hence reducing this latter component.

The second one, the N250, has a broader scalp distribution, somewhat larger over anterior sites than over posterior ones, and takes the form of a larger negativity to the target words that were unrelated to their preceding prime words than to target words that shared letters with their primes (Chauncey et al., 2008). Contrary to P150, this component is not elicited for individual letters or objects and is interpreted as the reflection of the processing of letters combinations (e.g., bigrams, trigrams), the mapping of prelexical form representations onto whole-word form representations (Grainger and Holcomb, 2009).

We suspect that many reasons could explain why we have not observed these two components in our results. First of all, our task is quite different from those previously reported, in the sense that it is a task consisting in searching information, in real reading conditions, and it usually takes a lot of time and words between the goal of the text and a target word. In the above mentioned tasks in which P150 and N250 were reported, there were a few hundred milliseconds between a prime word and a target word. Therefore, we can assume that the form of the prime word is much more present in memory than those of the words of the goal in our experiment. Moreover, two words established the goal in our experiment, which perhaps reduces the role of their form in solving the task, and gives a “supra” goal that corresponds to a semantic combination of the 2 words' meaning. Therefore, we assume that our task requires semantic processes above all, especially since morphologically related word forms are not necessarily associated with the same meaning. A measure of morpho-semantic coherence (Ford et al., 2003) captures the difference between semantically transparent and semantically opaque morphologically related words (Hauk et al., 2006). For example, “government” and “govern” present a cosine value (LSA) of 0.68 whereas that of “department” and “depart” is 0.04. We have some reason to believe that the processes partaking in our task are not related to the perception of the word form, but rather linked to deeper processes reflected by later components.

At the key event JustAfterTarget, we have found a first positive component synchronized with this event that is larger than the previous and subsequent ones. The effect is significant in the 50–90 ms window after the onset of the JustAfterTarget event. Reported at the onset of the TargetWord event (when the subject is supposed to read the target word), this effect appears in average in the 280–320 ms window (by adding the mean durations of one fixation and one saccade). Moreover, as explained before, the EFRP extracted at the key event JustAfterTarget is the sum of multiple contributions (Figure 13A): the expected response (blue line) is mixed with the previous responses, mainly from the TargetWord (red line) and JustBeforeTarget events (black line). More specifically, we suppose that the increase in positivity is due to the accumulation of a more positive P300 component elicited from the TargetWord fixation (a more positive P300 component is illustrated by a continuous red line as opposed to the red dotted line) to the early positive component P1 from the JustAfterTarget event (blue line).

As a reminder, a positive decision means that the target word has been found, the content has been retrieved and the decision to leave the text can be made. As seen in introduction, the P300 component can be divided into two subcomponents, P3a and P3b (Polich, 2007). While the former component originates from stimulus-driven frontal attention, the latter originates from a temporal-parietal activity associated with attention. Our P300 may be interpreted as a P3b component reflecting the decision to stop the search for information.

Indeed, many reports suggest that the amplitude of P3b varies according to the role that the eliciting stimulus plays in the participant's task. For instance, it has been shown that P300 is enhanced when the stimulus “resolves uncertainty,” is made “task relevant” or is novel or unexpected. A main hypothesis regarding the functional significance of the P3b is that it is related to decision-making (Verleger et al., 2005; Verleger, 2008), which generally results in the enhancement of the P3b amplitude when people are required to make decisions based on stimuli (Acosta and Nasman, 1992). Specifically, there is a relationship between the amplitude of P3b amplitude and the confidence in a decision, with a higher amplitude being associated with greater degrees of confidence (Andreassi, 2006). P3b has been regarded as a sign of memory access processes, evoked by the evaluation of stimuli in tasks that require some form of action (Kok, 2001). Decision-making refers to processes responsible for the identification of the presence or identity of task-relevant stimuli and the mapping of these onto appropriate responses (Nieuwenhuis et al., 2005), which is exactly the case of our target words. For instance, when equated for frequency of occurrence, target stimuli (i.e., stimuli requiring a response) typically elicit higher P3b amplitudes than non-target stimuli. Furthermore, the P3b component appears as an index of controlled processing resources allocated to decision-making or the updating of memory after a decision is made (Donchin and Coles, 1988). This is typically what our participants have to do after reaching the target word: to decide whether they continue reading. Unfortunately, when synchronized with the TargetWord event, this effect on the 280–320 ms window is not significant. Indexing decision-making, P3b seems to be spread out onto the next fixations. The absence of any effect on the target word may be explained by the fact that (1) the early components' jitter (± 65 ms, cf. Table 3, see Figure 13A) is not large enough to reduce this overlapping (blue line) and (2) the overlapping with the late component elicited from the JustBeforeTarget event is not that disrupting, as its amplitude is weaker and its shape in low frequency. We plan to carry on this work and use the Adjar algorithm (Woldorff, 1993) to evaluate overlapping before we extract more reliable EFRP components. However, at the current state of our research, it is not very clear cut if this increase of positivity interpreted as a possible P300 is really linked to a decision-making processes, or more simply to the processing of the significance of the word in the context of the task. In other words, it is possible that a participant decided to leave the text not specifically at the reading of a target word, but slightly later. Decision-making could be in this case a more global process of integration of information from the context (i.e., the previous words) and specific words (i.e., target words). To answer this question, one can extend this research by replicating this experiment in changing the instructions (e.g., in asking participants to read the whole text and making the decision at the end of the reading), in order to separate the decision related to the perception of the relevance of a word in the context of the task, from the decision to interrupt the reading of the text.

Another possible explanation of this increase in positivity might be linked to the increase in the amplitude of the P325 component. This component, whose posterior hemisphere distribution is more oriented to the right, confirms our results in that it is more positive to targets that overlapped their primes in all letter positions and more negative to unrelated and partially overlapping prime-target pairs (Chauncey et al., 2008). At the moment, we do not have enough elements to decide between these two explanations, namely an increase in amplitude of a P300 and/or a P325, even if, as mentioned above, the kind of task in which a P325 has been reported is quite different from ours. Fact is that our future experiments should draw a clear distinction between target words that are highly semantically related to the goal while having different verbal forms, and target words sharing the same verbal form that are semantically highly related to the goal. It would be interesting to observe, both at behavioral and electrophysiological levels, which kind of component would be elicited, and if one or other of these kinds of targets would result in the greatest acceleration in decision-making.

### Negative decision (incongruent) indexed by a N400?

Incongruent words were defined as words that had a low frequency and were unrelated to the goal. Again, behavioral data showed—as we expected—that the remaining number of fixations after a fixation on an incongruent word was significantly less important than the remaining number of fixations after any other fixation on a word other than an incongruent word, at the same rank of fixation. Thus, we can assume that the lack of a semantic link between the incongruent word and the goal has been well perceived and partakes in decision-making. From the electrophysiological point of view, this semantic mismatch is reflected by a specific component, the N400 component.

Our results show that the first positive component elicited by the Rank2AfterIncongruent fixations was less positive than on previous and subsequent fixations. This effect is significant in the 50–120 ms window after the onset of the Rank2AfterIncongruent event. Reported two fixations earlier, i.e., at the onset of the IncongruentWord event (when the subject is supposed to read the incongruent word), the effect appears in average in the 510–580 ms window (by adding the mean durations of two fixations and two saccades). Our hypothesis is that this reduction of the positive amplitude is due to an increase in the amplitude of the N400 component elicited from the IncongruentWord event occurring two fixations earlier. This increase is illustrated by the component in red line as opposed to the component in red dotted line in Figure 13. The main disrupted overlapping is due to the early components (green line) from the Rank2AfterIncongruent event, but here the jitter is larger (± 125 ms, cf. Table 3, see Figure 13B), and then the overlapping is less disrupted and the effect is experimentally significant in the 500–530 ms window, when EFRP is extracted at the onset of the IncongruentWord event. This late negative component elicited by the fixation on a incongruent word, with the same scalp distribution (parieto-central and occipital) as the early effect elicited by the second fixation after an incongruent word, was interpreted as an N400 component.

The functional signification of N400 is linked to meaning processing. More specifically, N400 is largest for semantic anomalies, with its amplitude inversely related to the degree of semantic relatedness of a stimulus event (see for a review Kutas and Federmeier, 2000). The N400 amplitude is also highly correlated with an offline measure of the word's expectancy (i.e., cloze probability), that is to say the percentage of individuals who would continue a sentence fragment with that word. Many studies have shown this predictability effect on the N400 component, with low predictability words eliciting a larger N400 component than high predictability words (Lee et al., 2012).

Our result is quite original, for very few studies have observed an N400 effect in such a “large” linguistic material, in the sense that incongruent words are specific to a completely different goal and that the goal was disclosed very early in the reading, even before the presentation of the text. This result is in line with recent theories on the role of the N400 component (Kutas and Federmeier, 2011), that interpreted it as an integration of the semantic information accessed from the current word with semantic information spread over multiple words (e.g., discourse message-level representations, presumably held in working memory). In this case, the N400 amplitude is more related to higher-level factors than to lower-level ones. The N400 component reflects a process that could pertain to discourse comprehension, in which the reader has to develop a mental representation of the text, which requires a continuous process of integration of the words presented in the text into background knowledge. However, we have to slightly nuance this conclusion, because the incongruent words, as we have defined them, may also be incongruent with respect to the context of the sentence they are taken from. In the future, a relevant distinction between the incongruent words that are incongruent only in relation to the goal (and not to the preceding context of the sentence) and those that are incongruent in their sentence and not relatively to the goal, would be most welcome.

### Conclusion

The aim of this experiment was to co-register EEG signals and eye tracking measures while participants had to decide stop or not reading. We found two early effects: a more negative component on the fixation *N* + 2 after an incongruent word and a more positive component on the fixation *N* + 1 after a target word. The first one was interpreted as a N400 component related to the processing of the incongruous word. Further experiments needs to be put in place to better explain the increase of positivity. The present paper demonstrates how EFRPs can be a useful tool for the identification of the cognitive processes at work during a natural task such as the search for information in texts. Indeed, the major benefit of the EFRPs technique used in our experiment is to investigate EEG components during free eye-movements, in ecological reading conditions. However, the major challenge this technique is still confronted with is to disentangle the overlapping processes occurring during a short fixation duration. The Adjar deconvolution technique could represent a partial solution to the problem and we are confident that future research will strongly benefits from cross-linking eye movements and ERPs. We hope that our paper also largely contributes to this issue by presenting precise methodological points.

### Conflict of interest statement

The authors declare that the research was conducted in the absence of any commercial or financial relationships that could be construed as a potential conflict of interest.
